# Highly selective and sensitive phosphate anion sensors based on AlGaN/GaN high electron mobility transistors functionalized by ion imprinted polymer

**DOI:** 10.1038/srep27728

**Published:** 2016-06-09

**Authors:** Xiuling Jia, Dunjun Chen, Liu Bin, Hai Lu, Rong Zhang, Youdou Zheng

**Affiliations:** 1 Jiangsu Provincial Key Laboratory of Advanced Photonic and Electronic Materials, School of Electronic Science and Engineering, Nanjing University, Nanjing 210093, P. R. China; 2 Chuzhou Vocational and Technical College, Chuzhou 239000, P. R. China.

## Abstract

A novel ion-imprinted electrochemical sensor based on AlGaN/GaN high electron mobility transistors (HEMTs) was developed to detect trace amounts of phosphate anion. This sensor combined the advantages of the ion sensitivity of AlGaN/GaN HEMTs and specific recognition of ion imprinted polymers. The current response showed that the fabricated sensor is highly sensitive and selective to phosphate anions. The current change exhibited approximate linear dependence for phosphate concentration from 0.02 mg L^−1^ to 2 mg L^−1^, the sensitivity and detection limit of the sensor is 3.191 μA/mg L^−1^ and 1.97 μg L^−1^, respectively. The results indicated that this AlGaN/GaN HEMT-based electrochemical sensor has the potential applications on phosphate anion detection.

Phosphate is one of the most important electrolytes and an essential component of all living organisms, as they take part in almost all metabolic processes^1^. A high phosphate level is detrimental to the environment and can lead to eutrophication which disrupts aquatic life cycles^2^,^3^,^4^,^5^,^6^,^7^,^8^,^9^. Phosphate levels is higher than 0.1 mg L^−1^, which may lead to many health concerns^10^,^11^, because moderate increase in phosphate concentration in natural water results in eutrophication and consequently lowers the dissolved oxygen concentration. Eventually, it will cause the death of certain fishes and other aquatic animals due to suffocation. While the normal phosphate level in the human body is higher, about 25–45 mg L^−1^ in blood serum^12^. It will be lead to the physiological and pathological changes of the human body when phosphate level is out of the normal range. So, the phosphate detection and recognition are of great significance for the controlling and guarding against the eutrophication and human disease occurrence.

Various detection strategies for phosphate have been developed including spectrophotometry^13^,^14^, fluorometry^15^,^16^, chromatography^17^. However, these methods are time consuming, laborious, require expensive instruments, well trained operators, and often employ potentially carcinogenic chemicals for the analysis^5^. The drawbacks of these techniques make them not easily to be used for on-line monitoring.

Recently AlGaN/GaN high electron mobility transistors (HEMTs) have been developed for favorable sensors, because the conducting two-dimensional electron gas (2DEG) channel is very close to the surface and extremely sensitive to adsorption of analytes^18^. In the past few years, development of ion selective monitoring such as potassium ions^19^, ammonium ions^20^, chloride ions^21^, mercury ions^22^,^23^,^24^,^25^ based on AlGaN/GaN HEMTs has attracted word-wide attentions. Due to excellent chemical and physical stability in water^21^,^27^, ion sensitive AlGaN/GaN HEMTs have showed great advantages in ion detection. Thus, it may be an excellent potential candidate for phosphate anion detection.

Molecular imprinting is a technology to create recognition sites in a macromolecular matrix using a template molecule^28^,^29^. Within molecularly imprinted polymers (MIPs), a large number of imprinted cavities designed for the template molecule are distributed, and these cavities are consistent to the template molecules in shape, size, and functional groups. So, MIPs not only have specific molecular recognition ability and high binding affinity for the template molecule, but also are described as artificial antibodies or receptors^29^,^30^,^31^. As the template is ion in the preparation process of MIPs, the synthetic products are called ion-imprinted polymers (IIPs)^32^,^33^,^34^,^35^,^36^. IIPs maintain all the advantages of MIPs, they can specially recognize the template ion. In the last few years, IIPs as selective sorbents for template ions have attracted much attention, and their applications in selective recognition, separation, and enrichment of ions as well as in the removal of toxic ions from aqueous medium for protecting the environment have been reported^37^,^38^,^39^,^40^.

In this work, we functionalized the ungated region of AlGaN/GaN HEMT sensor by phosphate anion imprinted polymers to detect and recognize phosphate anion. The current response showed that the fabricated sensor is very sensitive to phosphate anions, and meanwhile, it has no obvious response to other interfering anions, for example permanganate anion-sulfate anion and so on.

## Results and Discussion

**Figure 1:**
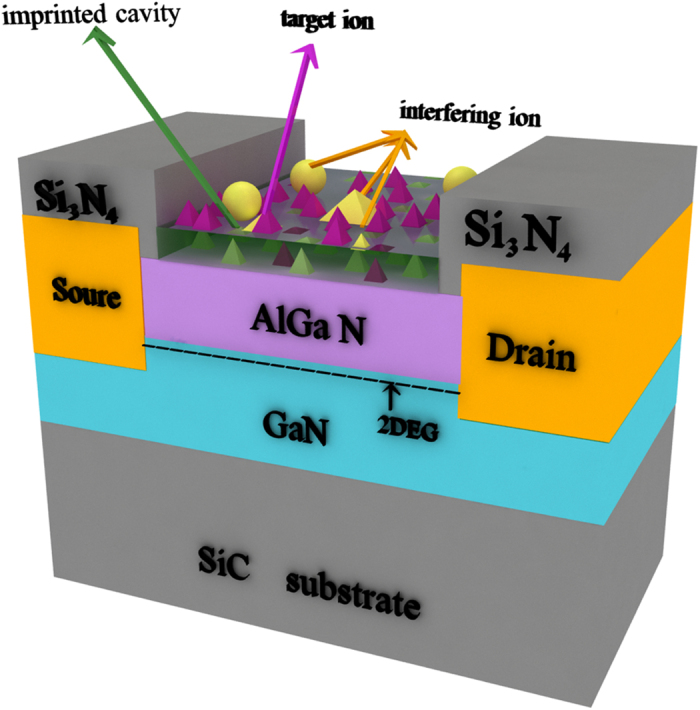
Schematic diagram of AlGaN/GaN HEMT sensor, the ungated area was functionalized with PO_4_^3−^ ion imprinted polymer.

**Figure 2:**
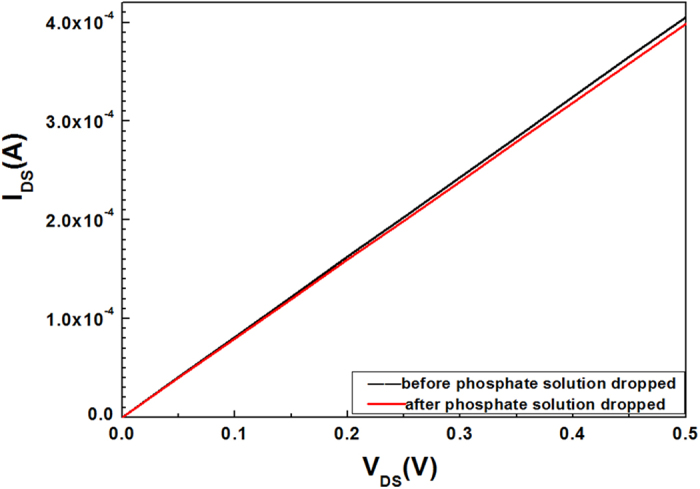
I-V characteristics of the ion imprinted AlGaN/GaN HEMT sensor before and after phosphate was dropped.

Figure 1 shows the structure diagram of the sensor. The current response was measured at room temperature using Cascade probe station. Figure 2 shows the voltage-current curves of the AlGaN/GaN HEMT-based sensor before and after dropping phosphate anion solution (PH = 5) onto the sensing region with a micropipette. We can observe that the source-drain current obviously decreases after dropping the phosphate anions. It is well known that the electrons in the 2DEG channel of undoped AlGaN/GaN HEMTs are induced by spontaneous and piezoelectric polarization charges. Any slight changes in the surface charge are transduced into a change in the concentration of the 2DEG in the AlGaN/GaN interface^41^. Therefore, the 2DEG concentration in the channel, which determines the source-drain resistance, is very sensitive to the change of surface charges of AlGaN/GaN HEMTs. When dropping the phosphate anion solution onto the functionalized surface of the AlGaN/GaN HEMT, phosphate anions with negative charges will occupy the imprinted caves in the sensing area due to the specific recognition and the electrostatic interaction between this template anion and the ion imprinted polymer. This process will decrease the surface potential (V_g_) on the ungated area of the AlGaN/GaN HEMT sensor, then leads to a reduction in the concentration of 2DEG in the channel, as described in following equation^42^ (1), and finally causes a reduced source-drain current.


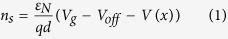


where ε_N_ is the permittivity of AlGaN, V_g_ is the gate voltage due to the surface states,

V_off_ is the threshold voltage, q is the electron charge, d is the total distance target ion to 2DEG, and V(x) is the channel potential.

**Figure 3:**
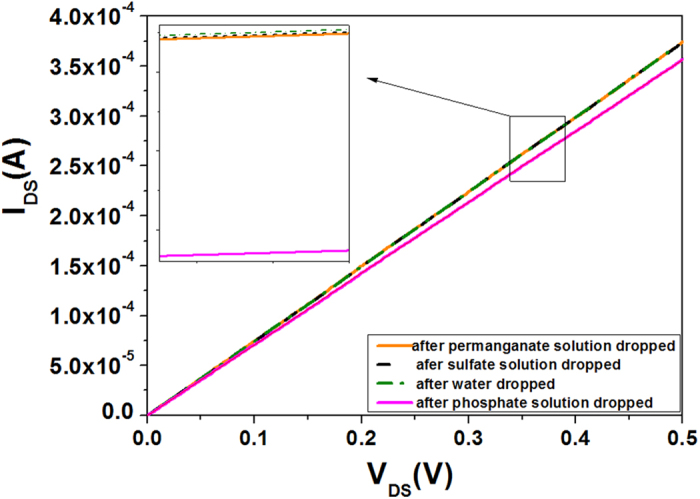
I-V characteristics of the AlGaN/GaN HEMT sensors for different anions detection, the inset is partial enlarged drawing.

However, when dropping permanganate and sulfate solution onto the sensing area, the source-drain current has almost no change as shown in Fig. 3, although permanganate and sulfate anions are of similar chemical structure to phosphate anion. This indicates that the sensor is of high selectivity for different anion solutions. This high selectivity can be attributed to the causes of specific recognition and electrostatic interaction between template ion and ion imprinted polymer. For the phosphate anion imprinted material, a great quantity of phosphate anion imprinted caves are distributed within the polymer layer on the sensing area of AlGaN/GaN HEMT. These caves are highly matched with phosphate anions in size and shape, which leads to the perfect recognition ability and strong binding action for phosphate anion^43^,^44^. In contrast, these imprinted caves are unmatched with MnO_4_^−^ and SO_4_^2−^ ion in size and shape, leading to basically non-recognizing and non-binding for two contrast anions. The order magnitude of the ionic radii are successively r_MnO4_^−^ > r_PO4_^3−^ > r_SO4_^2−^, so the imprinted cavities are small for MnO_4_^−^, and are big for SO_4_^2−^. Therefore, phosphate anion imprinted polymer exhibits a high selectivity to phosphate anion.

In addition, the temperature and pH value have effects on the current change of the sensors. The change of temperature can slightly affect the structural shrinkage and expansion of imprinted sites, and both tight and loose spatial structures of the imprinted sites will lead to the decrease in the recognition ability^45^. However, the change of temperature in a small range has little effect on current change according to our recorded experimental data. In our experiments, the current change was measured at room temperature which is consistent with the temperature that imprinted cavities formed, so the phosphate anions are matched with the imprinted cavities in size. Moreover, pH value of tested solution will affect the current change of the sensor. As pH < 4, phosphate predominantly exists as H_3_PO_4_ and H_2_PO_4_^−^, and H_3_PO_4_ species are hardly recognized by the ion imprinted polymers, so the current change of the sensor will decrease; As 7 < pH < 10, phosphate predominantly exists as HPO_4_^2−^ which is not matched with the imprinted cavities, and the sensor will be almost no response; As pH > 10, phosphate predominantly exists as PO_4_^3−^ and HPO_4_^2−^ which are also not matched with the imprinted cavities. As 4 < pH < 6, phosphate predominantly exists as H_2_PO_4_^−^ which is consistent with the template ion, and hence the sensor has the best response in this pH value range^43^,^46^. Therefore, both the functionalization of GaN-based HEMT sensor and experimental test are carried out at pH value of 5 in this work, and pH value was adjusted with HCl solution.

**Figure 4:**
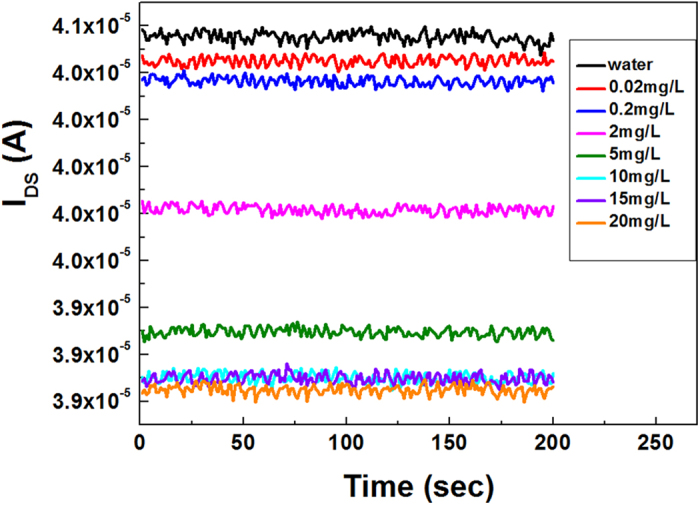
Current response of an AlGaN/GaN HEMT sensor to different phosphate concentrations from 0.02 mg L^−1^–20 mg L^−1^ at constant voltage of 50 mV.

Figure 4 shows the current response of the sensor to different concentration of phosphate anions at a constant voltage of 50 mV. Current is almost changeless when dropping water onto the sensitive area. In contrast to the water solution, when 0.02 mg L^−1^ of phosphate solution was dropped, an obvious current decrease was observed after the system reached a steady state. As expected, a gradual decrease in current was observed along with the increase of phosphate concentration in the measurement range from 0.02 mg L^−1^–20 mg L^−1^.

**Figure 5:**
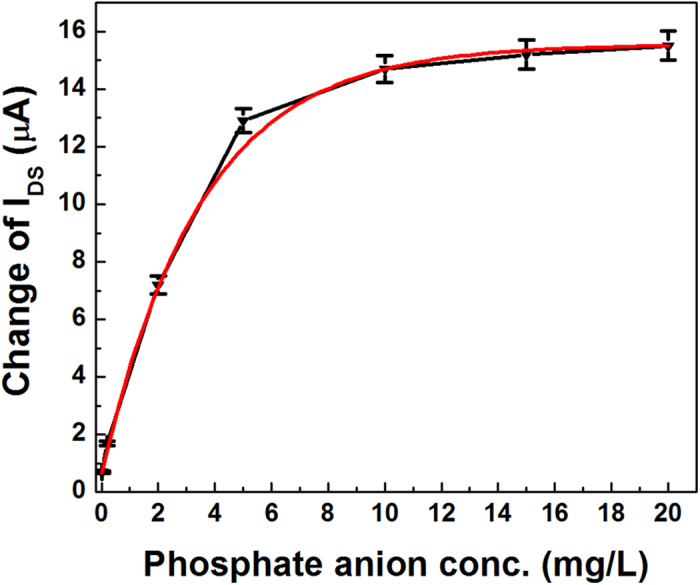
The current change versus the phosphate concentration at constant bias of 0.5 V. The data points and error bars represent the average value and the standard deviation, respectively, and each measurement was repeated at least six times.

In order to study the performances of the sensor, we analyzed the relations between current change and phosphate concentration. As shown in Fig. 5, the current change of sensor is approximate linear dependence on the target phosphate concentration in the range from 0.02 mg L^−1^–2 mg L^−1^. With further increase of phosphate anion concentration, the decrease of the current will be gradually saturated. It can be easily understood because the number of cavities in the ion imprinted polymer is limited. When phosphate anion concentration reaches a certain value, these cavities will be occupied completely, and the current change will also reach saturation.

The sensitivity is an important performance of the sensor, which was defined as the slope of the calibration line according to IUPAC. It can be observed that change of source-drain current exhibits an approximate linear dependence with phosphate concentration in the range of 0.02 mg L^−1^–2 mg L^−1^, and the linear regression coefficient of the calibration line is 0.993. Therefore, the sensitivity of the sensor is 3.191 μA/mg L^−1^. The detection limit is obtained using 3σ method (IUPAC)^47^.


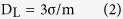


where σ is the standard deviation got from current method used in this experiment and m is the slope of calibration line. The detection limit is 1.97 μg L^−1^, which is better than the reported detection limits of 0.16 mg L^−1^ and 18.8 μg L^−1^ by Christopher^48^ and Gong^49^, respectively.

As the significant parameters of the sensor, the experimental reproducibility has to be investigated. Phosphate anions were detected for six independent experiments by using different AlGaN/GaN HEMT-based sensors. The maximum relative standard deviation (RSD) is 4.4% at the concentration level of 0.02 mg L^−1^, indicating acceptable reproducibility of the fabricated sensors. In addition, the fabricated sensor remained 98% of its initial current response after 15 days storage at room temperature, suggesting the satisfactory storage stability.

The AlGaN/GaN HEMT sensor functioned with ion imprinted polymer was fabricated successfully. It showed highly selectivity and recognition to phosphate anion and no response to other interfering anions, such as permanganate and sulfate anions. The sensitivity of sensor is 3.191 μA/mg L^−1^, and the detection limit is 1.97 μg L^−1^. Highly sensitivity and selectivity make the AlGaN/GaN HEMT-based sensor a promising application for phosphate anion on-line monitoring which may integrate easily the wireless microwave transmission function of HEMTs.

## Methods

### The growth process of AlGaN/GaN on SiC substrate and device preparation

Figure 1 shows the cross-sectional diagram of the sensor. The AlGaN/GaN heterostructure was grown by metal-organic chemical vapor deposition on a SiC substrate. A 3-μm-undoped GaN buffer layer was firstly grown on the substrate, followed by a 25 nm undoped Al_0.3_Ga_0.7_N layer was grown on the GaN layer to form the heterostructure. The two-dimensional electron gas (2DEG) is located at the interface between the undoped GaN layer and the AlGaN layer. Hall measurements showed a sheet carrier density of 1.85 × 10^13^ cm^−2^ and carrier mobility of 1650 cm^2^ v^−1^ s^−1^ at room temperature.

Mesa isolation was performed using an Inductively Coupled Plasma (ICP) etching system. Ohmic contacts were fabricated by depositing Ti/Al/Ni/Au (30/120/50/80 nm) layers using e-beam evaporation on the AlGaN layer and then annealing at 850 °C for 30 s in a flowing N_2_ atmosphere. The electrodes were protected to avoid direct contact to the solution by Si_3_N_4_ passivation layer (about 120 nm) deposited with plasma enhanced chemical vapor deposition. After that, photoresist (AZ5214) was used to encapsulate the source/drain regions, with only the ungated region open to allow the reactive ion etching to etch the surface. In order to reduce the surface damage induced by dry-etching, we adopted a method which combined virtues of dry-etching and wet-etching. Si_3_N_4_ passivation layer was etched 100 nm by dry-etching and the left was removed by wet-etching (HF:H_2_O = 1:10). At last, open ungated area was functioned with phosphate ion imprinted polymer.

### The surface functionalization of sensor

To modify the ungated region of sensor with the phosphate ion imprinted polymer, the treated AlGaN surfaces were oxidized, silanized and ion-imprinted successively^50^. During oxidizing step, the sensing surface was oxidized 40 s with Inductively Coupled Plasma (ICP). After oxidation, the samples were boiled in deionized water for 30 min at 100 °C and then blown dry by a nitrogen gun. 10% 3-aminopropyltrimethoxy- silane (AMPS, KH-540) solution was used to treat the samples for 24 h at 50 °C. In the steps of phosphate ion imprinting, the phosphate anion surface-imprinting was conducted in an aqueous solution system. Deionized water 50 mL, 0.6985 g of Na_3_PO_4_∙12 H_2_O as template, 1.5 mL of monomer methacryloyloxyethyl trimethyl ammonium chloride (DMC), and 0.328 g of cross-linker N,N’-Methylenebisacrylami-De (MBA) were added to a beaker, silanized AlGaN/GaN HEMTs were put into solution and N_2_ was bubbled for 30 min to exclude air. The pH value of the solution was adjusted to 5. The substance was heated to 35 °C, followed by adding 0.02915 g of initiator ammonium persulfate. The graft/cross-linking polymerization reaction was performed at 35 °C for 14 h. To remove the template phosphate anion, the device was fully washed with 2 mol L^−1^ NaCl solution. After washing away the template ions, a large numbers of phosphate anion imprinted caves will remain within this thin polymer layer on the sensing surface. Through vacuum drying, the AlGaN/GaN sensors functionalized by the phosphate anion imprinted polymers were obtained. (The chemical reaction processes of ion imprinting see supplementary information).

### Electrical measurements of the sensor

The current of source-drain was measured at room temperature using Cascade probe station (Instrument types: Tesla 200, Tungsten probe), Tungsten probes were pressed on the electrode of the source and drain during the measurements.

## Additional Information

**How to cite this article**: Jia, X. *et al.* Highly selective and sensitive phosphate anion sensors based on AlGaN/GaN high electron mobility transistors functionalized by ion imprinted polymer. *Sci. Rep.* **6**, 27728; doi: 10.1038/srep27728 (2016).

## Supplementary Material

Supplementary Information
