# Supplementary material for: Highly selective and sensitive phosphate anion sensors based on AlGaN/GaN high electron mobility transistors functionalized by ion imprinted polymer
Source: Sci Rep. 2016 Jun 9;6:27728. doi: 10.1038/srep27728 (PMC4899738; doi:10.1038/srep27728)
Supplement: Supplementary Information [file srep27728-s1.pdf]

Supplementary Information

Highly selective and sensitive phosphate anion sensors based on AlGaN/GaN high electron mobility transistors functionalized by ion imprinted polymer

Xiuling Jia[1,2], Dunjun Chen[1, *], Bin Liu[1], Hai Lu[1], Rong Zhang[1*] & Youdou Zheng[1]

[1]Key Laboratory of Advanced Photonic and Electronic Materials, School of electronic Science and Engineering, Nanjing University, Nanjing, 210093, P. R. China.

[2]Chuzhou Vocational and Technical College，Chuzhou, 239000, P. R. China.

**** Correspondence to D.J .C. (djchen@nju.edu.cn) or R. Z. (rzhangsdu@nju.edu.cn)***

The chemical reaction processes of ion imprinting：

a.

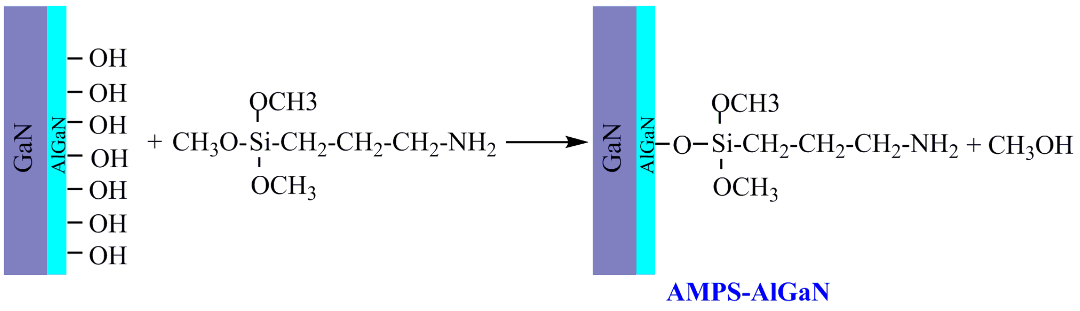

b.

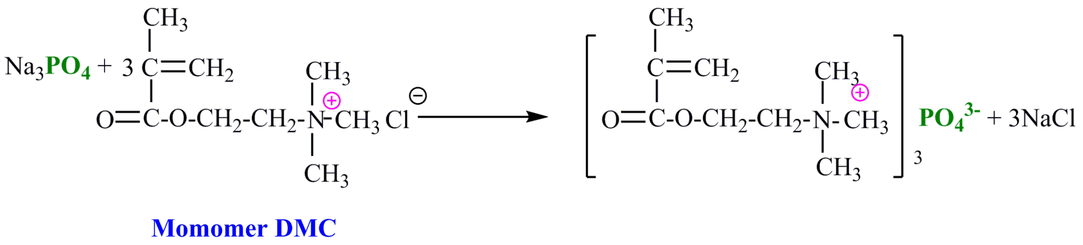

c.

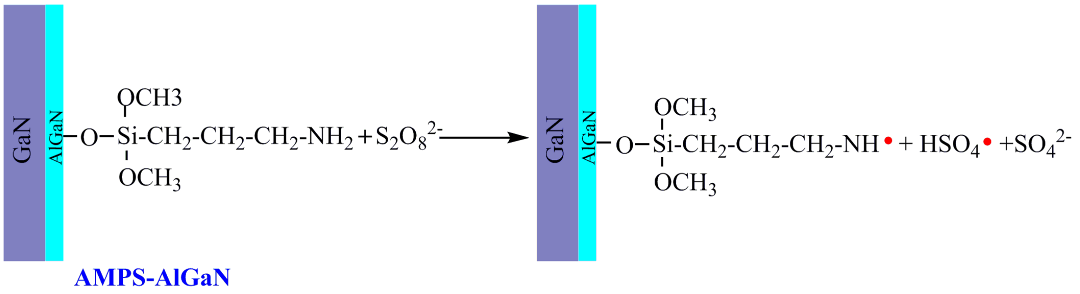

d.

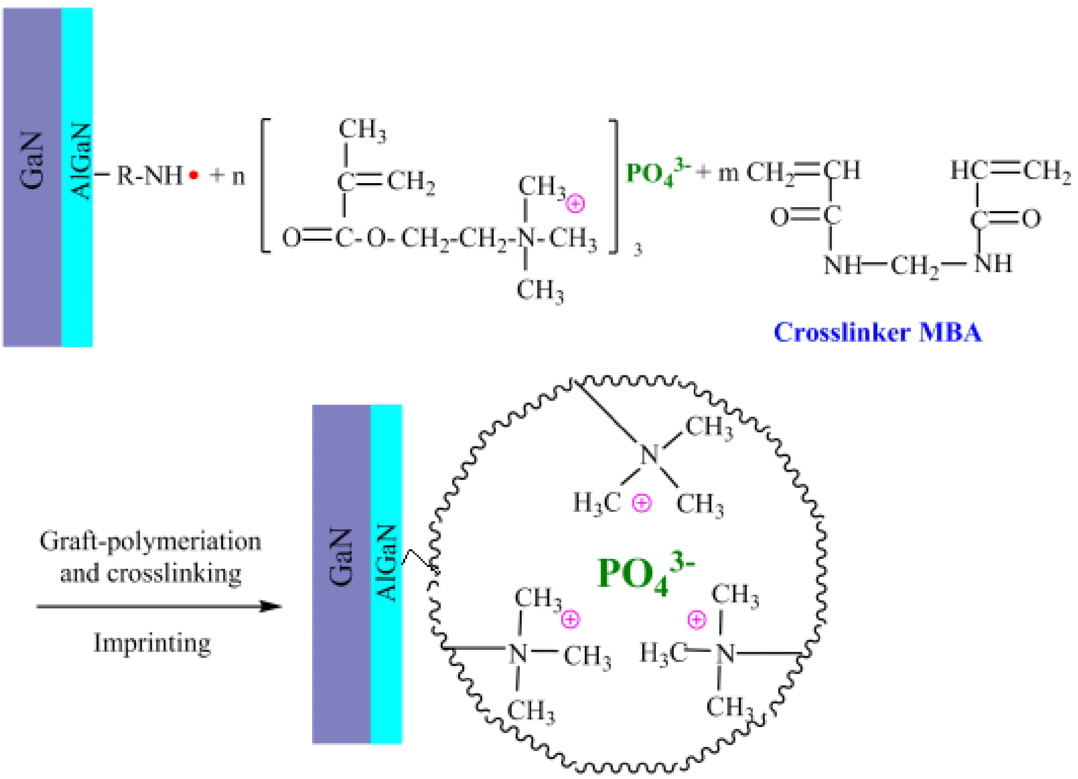

e.

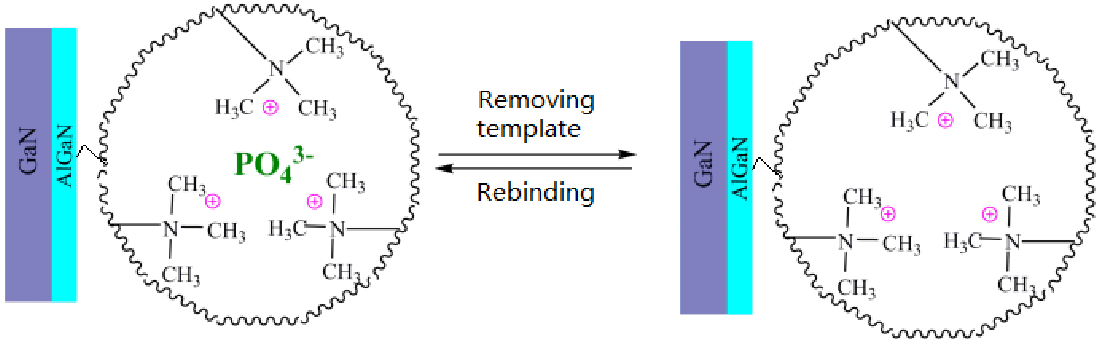

Figure 1. The chemical reaction processes of ion imprinting. **(a).** The sensor was first surface-modified with coupling agent AMPS. **(b).** Combination of monomer DMC with template PO_4^{3-} ion via ion exchange action. **(c).** Production of free radical on surfaces of modified silica gel particles. **(d).** Simultaneously surface-initiated graf-polymerizing and imprinting. **(e).** Removing template ion PO_4^{3-}.
